# Colonization kinetics and implantation follow-up of the sewage microbiome in an urban wastewater treatment plant

**DOI:** 10.1038/s41598-020-68496-z

**Published:** 2020-07-15

**Authors:** Loïc Morin, Anne Goubet, Céline Madigou, Jean-Jacques Pernelle, Karima Palmier, Karine Labadie, Arnaud Lemainque, Ophélie Michot, Lucie Astoul, Paul Barbier, Jean-Luc Almayrac, Abdelghani Sghir

**Affiliations:** 1 Institut de Biologie Intégrative de la Cellule, Université Paris Saclay, 91405 Orsay Cedex, France; 2 INRAE, PROSE, Université Paris-Saclay, 92761 Antony, France; 3 Genoscope, Institut de Biologie François-Jacob, CEA, Université Paris-Saclay, 91057 Evry, France; 4 Laboratoire SIAAP Site Seine Amont, Usine Marne Aval, 100 rue de la Plaine, 93160 Noisy-Le-Grand, France; 5 Génomique métabolique, Genoscope, Institut de Biologie François Jacob, CEA, CNRS, Université d’Evry, Université Paris-Saclay, 91057 Evry, France

**Keywords:** Microbiology, Environmental sciences

## Abstract

The Seine-Morée wastewater treatment plant (SM_WWTP), with a capacity of 100,000 population-equivalents, was fed with raw domestic wastewater during all of its start-up phase. Its microbiome resulted from the spontaneous evolution of wastewater-borne microorganisms. This rare opportunity allowed us to analyze the sequential microbiota colonization and implantation follow up during the start-up phase of this WWTP by means of regular sampling carried out over 8 months until the establishment of a stable and functional ecosystem. During the study, biological nitrification–denitrification and dephosphatation occurred 68 days after the start-up of the WWTP, followed by flocs decantation 91 days later. High throughput sequencing of 18S and 16S rRNA genes was performed using Illumina's MiSeq and PGM Ion Torrent platforms respectively, generating 584,647 16S and 521,031 18S high-quality sequence rDNA reads. Analyses of 16S and 18S rDNA datasets show three colonization phases occurring concomitantly with nitrification, dephosphatation and floc development processes. Thus, we could define three microbiota profiles that sequentially colonized the SM_WWTP: the early colonizers, the late colonizers and the continuous spectrum population. Shannon and inverse Simpson diversity indices indicate that the highest microbiota diversity was reached at days 133 and 82 for prokaryotes and eukaryotes respectively; after that, the structure and complexity of the wastewater microbiome reached its functional stability. This study demonstrates that physicochemical parameters and microbial metabolic interactions are the main forces shaping microbial community structure, gradually building up and maintaining a functionally stable microbial ecosystem.

## Introduction

The wastewater treatment process is based on the use of sludge microbial populations to treat domestic and industrial pollutants. These populations constitute a complex ecosystem with biomass concentration approximating 2–10 g L^−1^^1^, with the majority aggregated into structures called flocs. The floc structure represents a protection strategy for microorganisms against predation as well as toxic chemicals, meanwhile allowing efficient uptake of nutrients. These flocs may contain up to 10^10^ prokaryotes mL^−1^ and 10^6^ micro-eukaryotes mL^−1^. Molecular approaches reveal that they often share persistent prokaryotic and eukaryotic core species stably retained over time, including among others, members of the *Proteobacteria*, *Bacteroidetes*,* Firmicutes*, and *Actinobacteria* phyla^2–4^. However, in comparison with prokaryotes, micro-eukaryotic diversity has benefited relatively little from modern molecular tools and high throughput sequencing technologies. The few sequencing-based analyses performed on samples from WWTP or sewage treatment bioreactors suggest that large knowledge gaps in diversity and functional ecology of this group of microorganisms^5–8^ exist, preventing accurate definitions of their identity, diversity and their roles in the depollution process. To provide a holistic view of the functioning of whole ecosystems, major fundamental studies are necessary for assessing the network of interactions between all kinds of microbes (*Bacteria, Archaea*, *Eukarya* and viruses) and with their environment. This will permit the inference of the ecological rules guiding assembly of complex microbial communities and their functional implications across temporal gradients, and biological and physicochemical perturbations, which still await discovery. Such studies should help anticipate the structure and activity of microbial communities and consequently the functional stability of the ecosystem.

Recent studies have reported temporal variations in both composition and structure of microbial communities^9,10^. To the best of our knowledge, no work has studied the colonization kinetics and the establishment of the wastewater microbiome through to the constitution of a complex and functional microbial ecosystem. In the present study, we are taking advantage of the start-up of a domestic WWTP to achieve full characterization of 23 time series samples from the SM_WWTP over 236 days. We hence, followed the colonization kinetics of wastewater-borne microorganisms from March 3rd through October 31st, 2014, using high throughput prokaryotic 16S and eukaryotic 18S rRNA gene sequencing, until the establishment of a complex functional and stable ecosystem.

## Results

### Physicochemical and overall molecular diversity analyses of microbial populations

**Figure 1:**
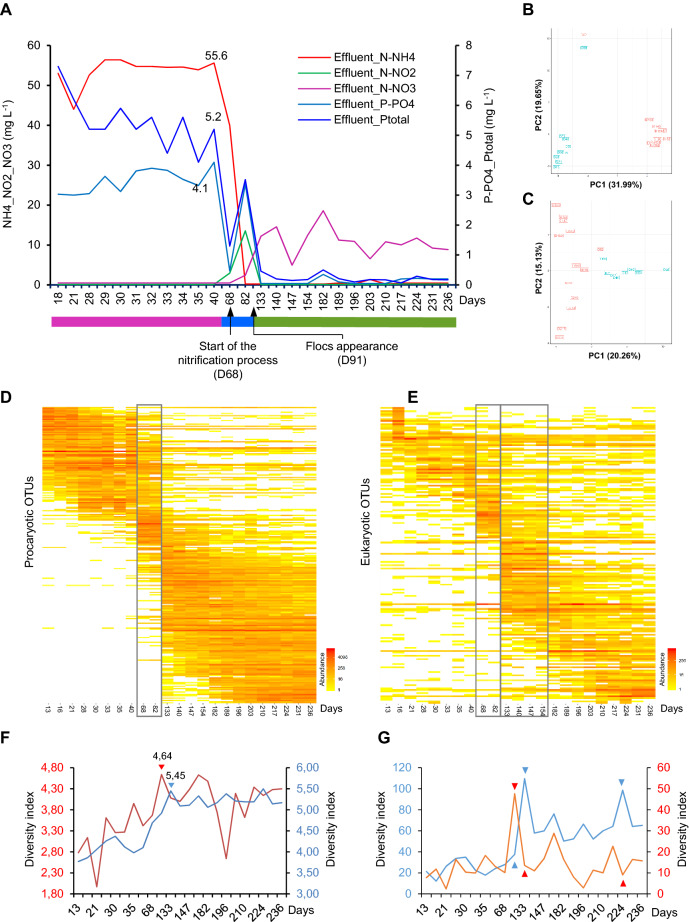
(**A**) The aerobic basin physicochemical parameter evolution through the 236 days. Blue scale color for P-PO4 and total phosphorus concentration only. Colored lines delimit the three periods of physicochemical parameter evolution: Pink represents the first period, blue, the intermediate period, and green represents the third period. (**B**) PCoA analysis of the 16S rDNA tags of the time series samples. The X and Y-axis explained 51.4% of the correlation between samples. PCoA separated the time series samples into three distinct groups constituting 3 phases. Phase 1, blue boxes (day 13–day 40); phase 2, blue and red boxes (days 82 and 68); and phase 3, red boxes (day 133–day 236). (**C**) PCoA analysis of the 18S rDNA tags of the SM_WWTP time series samples. The X and Y-axis explained 35.33% of the correlation between samples. (**D,E**) Hierarchically clustered heat map fingerprinting of the top 100 microbial OTUs respectively for bacteria (**D**), and eukaryotes (**E**), of the 23 time-series SM_WWTP samples. (**F,G**) Evolution of ecological diversity indices within the 23 time-series SM_WWTP. Scale colors: Red for *Eukarya* and blue for *Bacteria*. (**F**) Evolution of Shannon diversity indices within the SM_WWTP. (**G**) Evolution of Inverse Simpson diversity indices within the time series SM_WWTP. Scale colors: Red for *Eukarya* and blue for *Bacteria*.

The information regarding plant localization and process description, variation of plant operational parameters and physicochemical conditions, treatment performance, are detailed in Supplementary material online (Fig. S1, Fig. S2 and Table S1). Nitrogen measurement defines three periods: (1) The first period lasts for 11 weeks (13–40 days) during which ammonia in the aerobic basin is at its maximal concentration. (2) In the second period that lasts about 1 month, the ammonia starts decreasing concomitantly with the appearance of nitrite (day 40) and nitrate (day 68), until day 133 (Fig. 1A). (3) In the third period (133–236 days) nitrite is completely oxidized to nitrate at day 133 and remains relatively stable during the rest of the time. Biological dephosphatation started at day 40, and phosphorus concentration fluctuated from day 68 through day 133, to be completely reduced over this third period (133–236 days). Flocs decantation appeared 91 days after the start-up of the SM_WWTP.

Principal component analyses of the microbiota 16S and 18S rDNA data sets, revealed a diversification in microbial composition between samples, concomitantly occurring with the physicochemical transformations (e.g. nitrification, dephosphatation and floc development processes). This analysis indicates the constitution of three main homogeneous prokaryotic groups (Fig. 1B) whereas 18S rDNA tags indicates four eukaryotic groups (Fig. 1C). After stabilization, the plant could effectively remove 97.8% of chemical oxygen demand (COD) and 99.6% biological oxygen demand (BOD) from the sewage (Table S1).

A total of 584,647 and 521,031 high quality reads were generated from 23 SM_WWTP rDNA amplicon sequencing (Table 1), with an average of 25,419 and 22,653 reads per sample for bacteria and eukaryotes respectively. Phylotype richness calculation for individual samples based on the construction of rarefaction curves shows a complete saturation of microbial diversity (Fig. S3A, Fig. S3B). Sequence read clustering, based on 97% sequence similarity reveals, the occurrence of 6,696 bacterial operational taxonomic units (OTUs) and 1,423 eukaryotic OTUs (Table 1). Taxonomic affiliations using Silva database-132 shows that the 6,696 bacterial OTUs affiliated with 36 phyla among which we describe 30 persistent OTUs, occur at various abundancies throughout the study (Table 1). Among the 1,423 eukaryotic OTUs, we describe 19 persistent OTUs affiliating with 15 phyla (Table 1). Shannon and Inverse Simpson diversity indices show the occurrence of two periods, the first one where microbiota increase in numbers and diversity and the second period where the microbiota reaches its steady state (Fig. 1F,G).

**Table 1:** Summary of the SM_WWTP microbiome 16S/18S rRNA gene sequences and their affiliation at the phylogenetic domain level.

Domain	Number of reads	Number of OTUs
*Archaea*	306	18
*Bacteria*	584,647	6,696
Number of Phyla		36
Persistent OTUs^a^		30
*Eukarya*	521,031	1,423
Number of Phyla		15
Persistent OTUs^a^		19

^a^Persistent OTU: Any OTU who’s number of reads ≥ 1 sequence found in all samples.

### Overall description of wastewater microbiota phylogenetic groups

The most predominant phylogenetic groups within the *Bacteria* domain are the Gram-negative bacteria, represented by *Proteobacteria* and *Bacteroidetes*, and the Gram-positives represented by *Firmicutes* and *Actinobacteria,* averaging 64.8% and 13.1% of the total number of OTUs respectively. *Planctomycetes*, *Chloroflexi*, *Verrucomicrobia*, and *Acidobacteria,* represent an average of 17.6% of the total OTUs. The remaining 28 minor phylogenetic groups account for only 4.5%. In terms of abundance, nine phylogenetic groups, *Proteobacteria, Bacteroidetes, Planctomycetes, Chloroflexi, Firmicutes, Actinobacteria, Acidobacteria, Gemmatimonadetes,* and *Patescibacteria* (*Saccharibacteria*) represent up to 97.6% of the total reads (Fig. S5A), whereas the remaining 27 minor phyla made up only 2.4% of the total reads. Inside these phyla, the distribution of subphyla follows the same pattern, with major and minor subphyla (Fig. S5A). On the other hand, the distribution of genera abundance is characterized by a large diversity, i.e. many genera with a low abundance. However, some genera emerge with a slightly more elevated abundance; such is the case for *Acinetobacter,* an unknown genus from *Gammaproteobacteria, Terrimonas,* and an unknown genus from *Saprospiraceae,* and *Flavobacterium,* that together represent 20.6% of total reads (Fig. S5B)*.*

Fifteen phyla representing the *Eukarya* domain colonized the SM_WWTP. The most predominant in terms of percent of OTUs are *Nucletmycea, Holozoa, Amoebozoa, Rhizaria, Alveolata* (*Ciliophora, Apicomplexa*)*, Stramenopiles, Discoba, Chloroplastida,* and *Protalveolata*, representing an average of 89% of the total OTUs**.** Unknown and Multi-affiliation phyla make up 10% of the total OTUs. The remaining six minor phyla affiliate with *Apusozoa, Rhodophycea, Cryptista, Haptista, Metamonada,* and *Dinoflagellata.* Altogether, they totalize 1% of the OTUs. In terms of abundance, seven predominant phyla, *Holozoa, Rhizaria, Nucletmycea, Stramenopiles, Ciliophora, Discoba,* and *Amoebozoa,* represent > 98.77% of the total 18S rDNA reads. The remaining ten eukaryotic phyla made up only 1.23% of the total eukaryotic reads (Fig. S6A). Inside these seven predominant phyla, the subphyla follow the same distribution, with major and minor subphyla (Fig. S6A). In contrast to prokaryote genera distribution, eukaryotes are characterized by seven predominant genera: *Rhogostoma, Phascolodon, Mallomonas, Petalomonas,* an unknown genus from *Rhinosporideaceae,* an unknown genus from *Pseudoscorpiones*, and an unknown genus from *Adinetida,* that represents 56.65% of the total reads, not including *Cryptomycota,* a poorly known phylogenetic group (Fig. S6B).

### Colonization kinetics and phylogenetic diversity of microbial populations

Overall, temporal changes in the phylogenetic composition and abundancy of OTUs show a continuous increase during the first period. The number of eukaryotic and bacterial phyla reached their maximum at day 140, and 154 respectively (Fig. S4). However, Shannon's and inverse Simpson diversity indices show that *Eukarya* and *Bacteria* reached their greatest diversity between day 82 and day133 respectively (Fig. 1F,G). We show a net growth dependency between bacteria and eukaryotes, as is shown by the respective maximum and minimum percent of total phyla and OTU fluctuation in the time series samples (Fig. S4). If we take into account the evolution of physicochemical parameters (Fig. 1A), PCoA (Fig. 1B), and heat maps (Fig. 1D), all of them indicate a clear distinction between three bacteria profiles. The period from day 13 through day 40 represents the first profile; the intermediate profile is represented by the period from day 40 through 133; and the third profile is represented by the period from day 133 through day 236. However, PCoA and heat maps analyses show the distinction of four eukaryotic profiles (Fig. 1C,E). Figure 1D,E, illustrate the microbial community profile at OTU level in order to better assess the differences in microbial community composition in the SM_WWTP time series samples. As indicated in both figures, remarkable dissimilarities at both microbial composition and relative abundances were observed.

Canonical correspondence analysis (CCA) of the relation between bacterial community compositions and physicochemical properties of the time series wastewater samples showed that physicochemical properties such as COD, BOD, PO4, N-NH4, NTK correlated to the first phase predominant bacterial communities while nitrite and nitrate remarkably correlate to the second and the third phase bacterial communities respectively (Fig. S4B).

### Bacterial colonization kinetics at the phylum level

**Figure 2:**
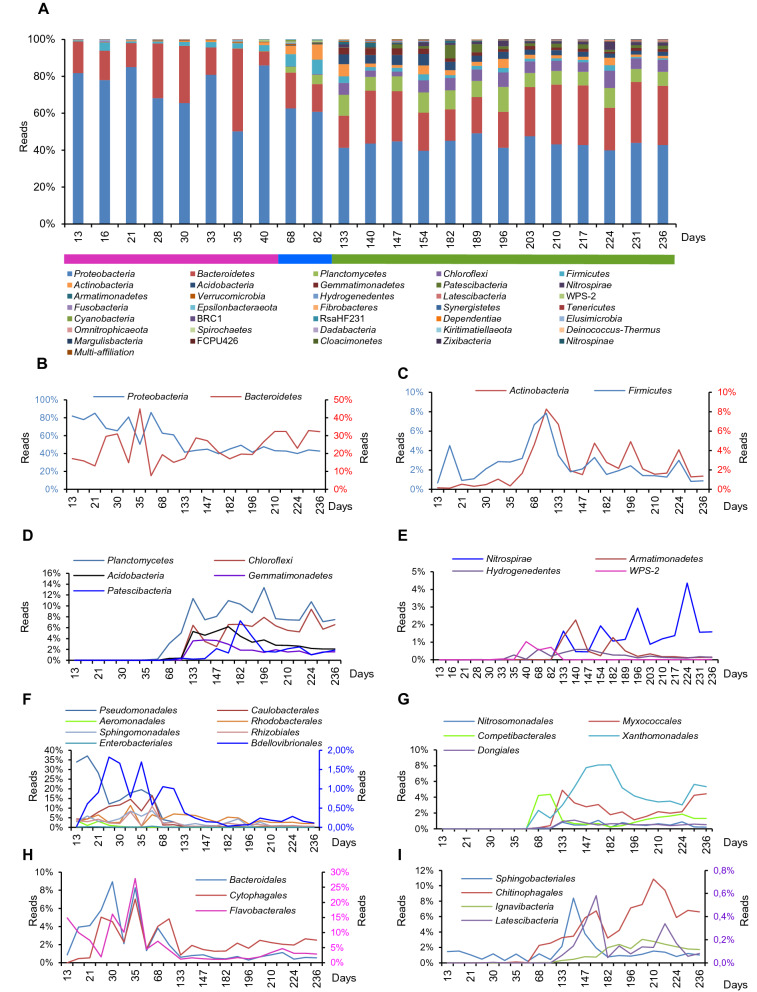
Relative abundance expressed as percent of total 16S rDNA reads and colonization kinetics of the main bacterial phyla over 236 days of the SM_WWTP colonization. (**A**) Relative abundance (%) of bacteria at the phylum taxonomic level within the 23 time-series SM_WWTP samples. Nine phylogenetic groups (in bold) represent up to 97.6% of the total reads. Colored lines delimit the three periods of bacterial evolution. Pink represents the first period, blue, the intermediate period, and green represents the third period. (**B**) Colonization kinetics of *Proteobacteria* and *Bacteroidetes*. Scale color: blue for *Proteobacteria*; red for *Bacteroidetes*. (**C**) Colonization kinetics of *Actinobacteria* and *Firmicutes*. Scale color: blue for *Firmicutes*; red for *Actinobacteria*. (**D**–**G**) Colonization kinetics of the remaining major bacterial phyla representing the late colonizers (**D,E**), and the first and late *Proteobacteria* colonizers at the phylogentic order level, in (**F,G**). (**H**,**I**) *Bacteroidetes* colonization kinetics at the phylogenetic order level over the 236 days of microbiota evolution within the SM_WWTP. The first colonizers (**H**) and late colonizers (**I**). Y-axis scale color: Green, for *Flavobacteriales* only, pink, for *Latescibacteria* only.

Throughout the study, the Gram-negative bacteria, *Proteobacteria* and *Bacteroidetes*, made up an average of 79.1% [59–99%] of total 16S rDNA sequences and to lesser extent the Gram-positive bacteria; *Firmicutes* and *Actinobacteria* represent an average of only 4.9% [1–16%] (Fig. 2B,C). However, the four phyla represent the most predominant prokaryotic populations present during the first and the second period of colonization (Fig. 2A), where *Proteobacteria* and *Bacteroidetes* together made up between 93 and 99% of the total bacterial sequences while the Gram-positives made up between 1 and 5%. Gram-negative and Gram-positive population abundance evolution throughout the study is quite different. While the *Proteobacteria* and *Bacteroidetes* evolve in an antagonistic way, *Firmicutes* and *Actinobacteria* evolve simultaneously and show the same growth trends (Fig. 2B,C). The appearance of a radiation of bacterial phyla affiliated with *Planctomycetes, Chloroflexi, Acidobacteria, Gemmatimonadetes, Patescibacteria, Nitrospira, Armatimonadetes,* (Fig. 2D,E) occurs over the second period, (starting from day 35 through day 133). Their appearance represents the beginning of the second phase of colonization.

### Bacterial colonization kinetics at the order level

Within the *Proteobacteria* phylum, two waves of bacterial phylogenetic groups are distinguished. The first wave of proteobacterial colonizers is composed of nine orders: *Pseudomonadales, Caulobacterales, Aeromonadales, Rhodobacterales Sphingobacterales, Rhizobiales, Burkholderiales, Enterobacteriales,* and *Bdellovibrionales.* They reach their maximal growth rate during the first period of colonization, but their abundance decreases to a minimum level over the second period. They maintain their presence at a very low population level or disappear from the WWTP (Fig. 2F). *Nitrosomonadales, Myxobacteriales, Competibacterales, Xanthomonadales,* and *Dongiales* represent the second wave (Fig. 2G).

Within the *Bacteroidetes–Cytophaga–Flavobacter* group, *Bacteroidales, Cytophagales,* and *Flavobacteriales* orders represent the first wave of colonizers over the first period (Fig. 2H). *Sphingobacteriales, Chitinophagales* and two minor orders, *Ignavibacteriales* and *Latescibacteriales* represent the second wave of colonizers (Fig. 2I). For both the *Proteobacteria* and *Bacteroidetes* orders, the second wave of colonizers appears 40 days after the start-up of the SM_WWTP.

### Bacterial colonization kinetics at the genus level

**Figure 3:**
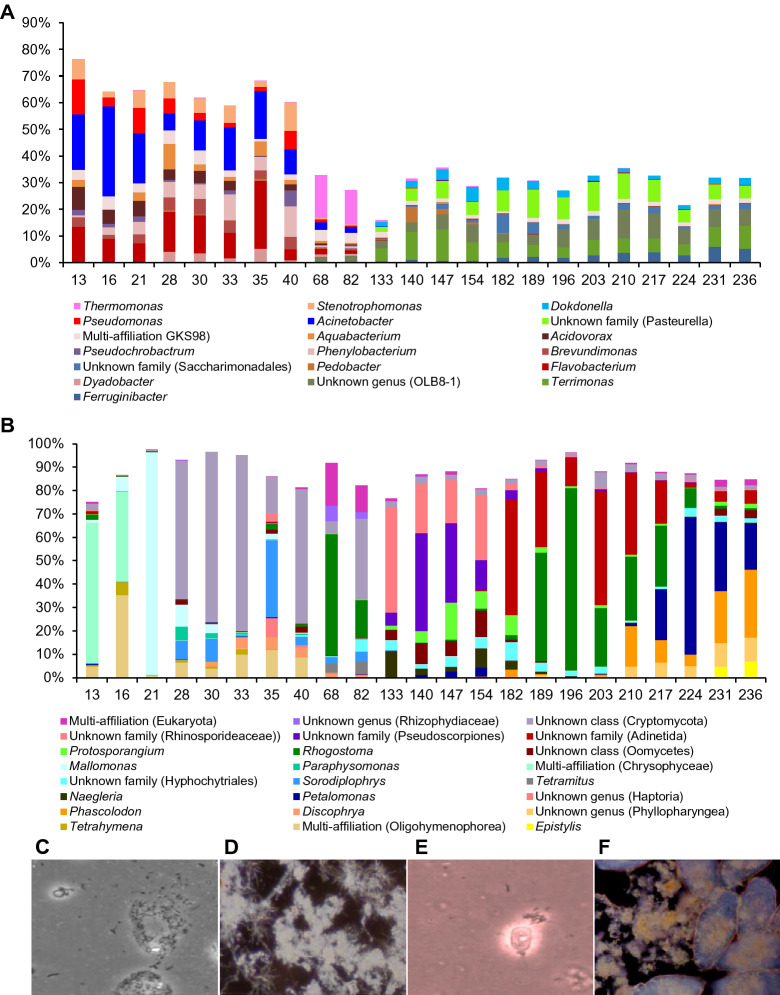
Relative abundance and kinetics of abundant bacterial (**A**) and eukaryotic (**B**) genera of the 23 time-series SM_WWTP samples. Genera were filtered for those that display an abundance rate ≥ 5% in at least one of the 23 samples. (**C–F**) Sludge microscopic images illustrating the structure of the SM_WWTP microbiota over the 236 days of time series sampling. (**C**) Bright-field microscopy image from day 40 sample (first phase) × 100. The image shows bacterial cells starting to organize into colonies and a small a free-living protist swimming beside them. (**D**) Dark-field microscopy image from day 230 sample (third phase) × 100. (**E**) Bright-field microscopy image from day 40 sample (first phase) × 400. The image shows a small free-living protist swimming among dispersed bacterial cells. (**F**). Dark-field microscopy image from day 230 sample (third phase) × 100.

Abundant genera kinetics, which appears with at least a 5% abundance rate in at least one of the 23 time-series samples, is presented in Fig. 3A. These abundant genera constitute around 60% of the total reads during the first phase, and decrease to about 25% in the second and third phase. This means that the global bacterial diversity increases at the end of the first phase. This increase takes place with a radical change of the genera (Fig. 3A; Tables S2, S3 and S4). Over the first period (13–40 days), we show an important population of sequence reads, (25–71%), predominantly affiliated with, *Pseudomonadales*, *Rhodobacterales*, *Caulobacterales*, *Sphingomonadales*, *Rhizobiales*, and *Enterobacteriales*, (Fig. 2F). The four top OTUs over this period affiliate with *Acinetobacter* (*Pseudomonadales*) 13.4%, *Phenylobacterium* (*Caulobacterales*) 5.7%, *Acidovorax* (*Burkholderiales*) 4.2%, and *Stenotrophomonas* (*Xanthomonadales*) 4% (Table S2). Over the second period (40–133 days), two OTUs stand out, affiliating with *Thermomonas* (*Xanthomonadales*) 13.8% and *Candidatus Competibacter* (*Competibacterales*) 2.9% (Table S2). The third period (133–236 days) is characterized by one leading OTU, PLTA13 (*Xanthomonadales*) 6.5% (Table S4). We notice an evolution of the structure of the wastewater microbial community, from dispersed planktonic bacterial and eukaryotic cells (Fig. 3C,E) to well structured and sessile bacterial and eukaryotic cells within a floc, as it is shown in Fig. 3D,F respectively.

### The nitrification–denitrification process

Physicochemical parameters show that ammonia concentration varied between 53.03 and 55.6 mg L^−1^ over 40 days, and starts decreasing from day 40 through day 82, ending up with 0.23 mg L^−1^ at day 82. At the same time, we notice an increase of nitrite and nitrate concentrations at day 40 and day 68 respectively (Fig. 1A). After that, nitrification–denitrification processes remain stable over the rest of the time.

### Colonization kinetics and phylogenetic diversity of the Eukaryotic population

**Figure 4:**
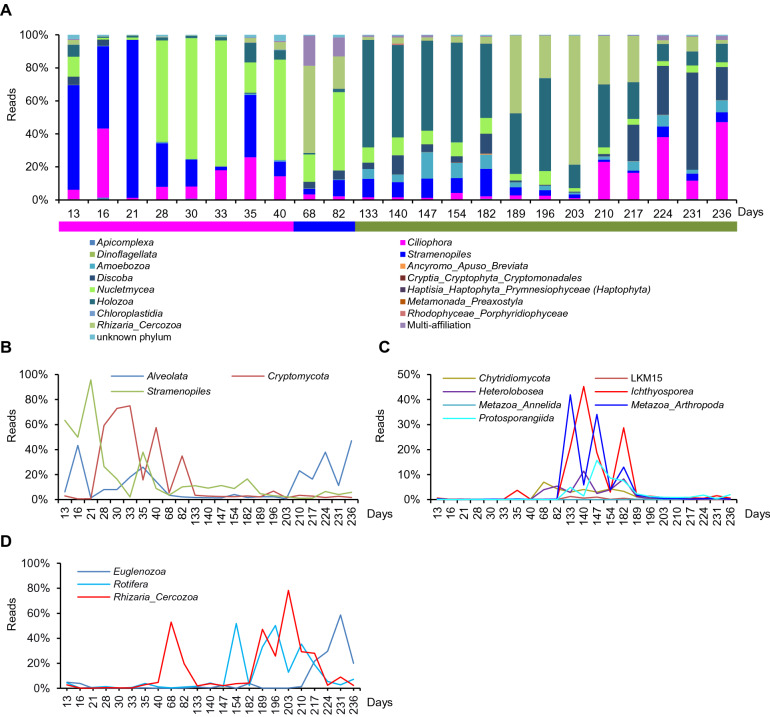
Relative abundance expressed as percent of total 18S rDNA reads, and colonization kinetics of the main eukaryotic phyla over the 236 days of the SM_WWTP colonization. (**A**) The relative abundance evolution of the 15 eukaryotic phyla, expressed as percent of total 18S rDNA reads over the 236 days of SM_WWTP colonization. Colored lines delimit the three periods of eukaryotic evolution. Pink represents the first period, blue, the intermediate period, and green represents the third period. (**B**) Curves showing the evolution of the first eukaryotic colonizers, (**C**) the intermediate eukaryotic colonizers, and (**D**) the late eukaryotic colonizers.

Implantation kinetics of the major eukaryotic organisms (Fig. 4A) shows three types of populations, the first colonizers represented by *Nucletmycetea* (*Cryptomycota*), *Stramenopiles* (*Ochrophyta*), and *Alveolata* (*Apicomplexa,* and *Ciliophora*) (Fig. 4B). Intermediate colonizers, which started colonizing the SM_WWTP after 40 days, are represented by *Nucletmycetea* (*Chytridiomycetes*, LKM15), *Heterolobosea, Ichthyosporea, Amoebozoa* (*Protosporangida*), *Metazoa* (*Arthropoda, Annelida, Protosporangea*), (Fig. 4A,C). The last group composed of *Rhizaria* (*Cercozoa*), *Rotifera*, and *Euglenozoa* (Fig. 4D), represents the late colonizers and appears after day 140. The same pattern is observed for minor eukaryotic phyla.

Kinetics of abundant eukaryotic genera, present at a rate of abundance > 5% at least once in the time series samples, are shown in Fig. 3B. They are representing about 85% of the total reads, and remain stable during the time series, in contrast to bacterial genera (Fig. 3A). This pattern is not fundamentally different from the pattern of eukaryotic phyla kinetics (Fig. 4A). This may be linked to the fact that phyla are represented mostly by abundant genera. However, there is also a radical change between the first and the third phase of the time series, as the first phase genera are replaced by novel abundant genera.

### Persistent prokaryotic and eukaryotic populations

Exploration of OTU occurrence shows the persistence of 30 and 19 OTUs for both prokaryotic and eukaryotic populations respectively (Fig. S7A, Fig. S7B). These OTUs consist of a minimum of one read but display variable abundances throughout the study. These persistent OTUs made up merely 0.004% of the total bacterial OTUs; they are affiliated with only four phyla: *Bacteroidetes, Proteobacteria, Firmicutes* and *Actinobacteria* (Fig. S7A), but accounted for an average of 14% [3–35%] of all 16S rDNA sequence reads. Although, the 19-core eukaryotic OTUs amounted to only 0.013% of the total OTUs, they accounted for an average of 69% [22–98%] of all 18S rDNA reads. They are affiliated with nine phylogenetic groups: *Holozoa* (*Arthropoda*, *Rotifera*), *Nucletmycea* (*Ascomycota*, *Cryptomycota*), *Rhizaria* (*Cercozoa*), *Alveolata* (*Ciliophora*), *Euglenozoa*, *Stramenopiles* (*Ochrophyta*, *Hyphochytriomycetes*, *Peronosporomycetes*), *Ichthyosporea*, *Heterolobosea* and unknown phylum (Fig. S7B).

## Discussion

Most ecological studies nowadays describe microbial communities from a perspective of phylogenetic levels, from phyla to genera, or based on species living within complex ecosystems. In microbial ecology, simple observation and description of community’s patterns are not enough, and there is a need to seek the mechanisms that may explain the occurrence of these patterns. A huge gap still exists in understanding how environmental factors and microbe-to-microbe interactions are shaping communities in space and time within complex microbial ecosystems.

In the present study, we have tracked microbial species and communities over a period of 236 days in a context where they were sequentially colonizing and building up a stable and efficiently functioning ecosystem. With this objective, we have sampled the aerobic basin of the SM_WWTP sequentially from March through October 2014. To obtain a holistic view of the process, we tried to be as complete as possible by analyzing both physicochemical parameters and microbial components (*Eukarya* and *Bacteria*) starting with the first inoculation of the basins with raw sewer microbes through the establishment of a complex, stable and functional microbial community.

When they first arrive in the basin of the SM_WWTP, sewer microbes still diluted in the black water, constitute the planktonic phase of the influent inoculum. They start building a robust food web composed of microbial communities well adapted to the available nutrients and the physicochemical parameters of the milieu. They start developing multiple physical and metabolic interactions between each other and their habitat, modifying and adjusting these parameters to make and optimize their own living. We cannot explain the colonization kinetics without taking into account the physicochemical parameter evolution and the network of interactions between microbial species over the overall colonization period.

### The nitrification–denitrification process

Classically, in complex and functionally stable wastewater ecosystems, ammonia oxidation is achieved by species affiliated with *Nitrosomonas europaea, Nitrosomonas eutropha, Nitrosococcus, Nitrosovibrio, Nitrosospira, Nitrosoarchaeum, Nitrosocaldus, Nitrosopumilus, Nitrososphaera,* and *Brocadia*^11,12^. However, our study shows that none of these genera was detected over the first period. The abrupt decrease in ammonium concentration in the aerobic basin during the intermediate phase (40–33 days) may be explained in part by the metabolic activity of the heterotrophic nitrifiers and denitrifiers, and also, by the assimilation of nitrogen by the microbiota, which we have shown to increase and diversify over the first and the intermediate period. Literature examination indicates that microorganisms such as many prokaryotes and fungi carry out heterotrophic nitrification and denitrification^13^. Fungi, such as *Aspergillus flavus*^14^, *Verticillium* sp.^15^, *Absidia cylindrospora*^16^, also carry out heterotrophic nitrification. It has been demonstrated that isolated fungal strains can remove both nitrogen and phosphorus from wastewater^17,18^. Liu et al*.,* showed that the concentration of ammonia in chicken manure was significantly lowered compared to the control group using the fungus *Paecilomyces variotii*, they isolated from the same environment^19^. By studying competition between the heterotrophic nitrate bacterium *Paracoccus denitrificans* and *Nitrosomonas europaea* (autotrophic bacterium)^20^, the authors have shown that under limiting oxygenation conditions and for high C/N concentration, bacterial heterotrophic nitrification becomes the dominant process and reaches up to 60% of total nitrification^21^. Moreover, numerous studies show that denitrification is usually carried out by facultative anaerobes such as gram-negative classes of α-, β- and γ-*Proteobacteria* (e.g. *Acinetobacter calcoaceticus, Alcaligenes faecalis, Microvirgula aerodenitrificans, Paracoccus denitrificans, Thiobacillus denitrificans,* and the genera *Acidovorax*, *Agrobacterium, Azoarcus, Thauera, Hyphomicrobium, Methylobacterium, Rhodobacter,* or the families *Pseudomonadaceae, Comamonadaceae*, and *Rhodocyclaceae*^22–29^. Meanwhile, many diazotrophic microorganisms, which fix atmospheric nitrogen gas into a more usable form, such as some species of *Azospirillum* and *Bradyrhizobium*, are able to denitrify^30^. Denitrification is also found among a few *Archaea*^31,32^ and *Fungi*^33,34^, including *Ascomycota* (e.g., *Fusarium oxysporum*, *Fusarium solani, Cylindrocarpon tonkinense* and *Gibberella fujiuroii*) and *Basidiomycota* (e.g., *Trichosporon cutaneum*). The fungi *Fusarium oxysporum* and *Cylindrocarpon tonkinense* constitute veritable denitrifiers. This denitrification was confirmed by observations showing that nitrate and nitrite reductions occurs in the mitochondria^35^. On the other hand, it has been demonstrated that these operations are coupled to a net synthesis of ATP^36^. Denitrification has also been described in some foraminifera^37^.

In the present study, sequences affiliated with above orders, families or genera involved in heterotrophic nitrification–denitrification are present at high abundance within the first phase of the time series samples. They accounted for between 19 and 95% of the total sequence reads during the first phase and only between 6 and 14% over the second phase. We think that, the nitrification–denitrification process may be predominantly triggered by heterotrophic prokaryote and eukaryote components of the ecosystem over the first period.

During the intermediate and the third phase (40–236 days), we show a gradual appearance of functional groups not present before, or present, but at a low level, such as, the nitrifying-denitrifying lithoautotrophic bacteria, hydrolyzing bacteria, and phosphate accumulating bacteria. Autotrophic nitrifying bacteria are usually associated with the flocs as expected from these organisms that grow in tight micro-colonies embedded in sludge flocs in order to perform their specific function, joined by the denitrifiers in tight association^38^. Ammonia oxidizing bacteria affiliated with either *Nitrosospira* or *Nitrosomonas* genera and some gram-positive bacteria such as *Bacillus licheniformis, Paracoccus denitrificans* have been shown to be capable of denitrification^39–43^. Candidatus Accumulibacter is a genus affiliating with polyphosphate-accumulating organisms which uses nitrite as electron acceptor for denitrifying phosphorus removal^44^. This finding is in line with recent reports showing that N_2_ production by denitrification is higher in particulate matter than in the planktonic phase in both marine and river environments, correlating with the organic content of the particles^45^. In our study, the autotrophic nitrifying-denitrifying bacteria were undetectable during the first planktonic phase, where aggregates or flocs were still under formation. The autotrophic ammonia and nitric oxidizers appear starting from day 82 (Figs. 1A, 2E).

### Exopolysaccharide secretion and flocs formation

Microorganisms take advantage of the nutriment-rich wastewater to synthetize reserve substances, among which, the exopolysaccharides (EPS) and organize species-rich structures in the form of microbial aggregates and planktonic biofilms called flocs. In our study, we observe that microbial populations are gradually switching from mostly planktonic to a more aggregated sessile life. The appearance of well-structured flocs requires between 2 and 3 months of adaptation and acclimation to the novel habitat, and provides stability and durability of metabolic interaction within the ecosystem. Hence, microscopy images presented in both, Fig. 3D,F show well-structured microbial cells, where bacteria and sessile protozoa (probably *Vorticella*), are tightly attached within the floc matrix. Our finding is in good agreement with studies showing a constant enrichment of EPS producing microorganisms over the first period. This period favors biomass structuration that supports process efficiency and stability. Microbial EPS are an abundant and important group of compounds that can be secreted by *Archaea*, *Bacteria*, *Fungi* and Algae^46–48^. EPS protect bacteria from environmental stresses, tolerate higher concentrations of many biocides, and play a definite role in sludge flocculation, that helps microorganisms efficiently biodegrades a wider range of substrates than pure cultures of free-living microorganisms. Within EPS, microorganisms can establish stable arrangements and function as synergistic micro-consortia such is shown in Fig. 3D,F, enabling the cells to function in a manner similar to multicellular organisms and complement each other’s functions. The EPS matrix facilitates nutrient sequestration, the retention of exo-enzymes, cellular debris and genetic material. It can be considered as a microbial recycling yard^46^. Moreover, EPSs are of considerable importance in the removal of pollutants from wastewater, in bio-flocculation and settling and in the sludge dewatering. *Bacteria* affiliated with orders and families of *Firmicutes* (*Leuconostocaceae* and *Streptococcaceae*), and *Proteobacteria* (*Burkholderiales*, *Pseudomonadales* and *Xanthomonadales*), among others have been described as producers of polysaccharides^49,50^.

Within the transitional period (40–133 days), *Acinetobacter* represents only 2.2% of dominant genera; it is gradually replaced by novel OTUs affiliated namely with *Thermomonas*, 13.8%, Candidatus Competibacter 2.9%, and *Arenimonas* 2%, etc. (Table S2). This intermediate phase composed of 371 persistent OTUs, making up to 76% of the total reads, with a predominant bacterial composition completely different from the first period or the intermediate period.

The third phase is marked by the settlement of novel functionally major colonizers such as *Planctomycetes, Acidobacteria, Patescibacteria, Chloroflexi, Gemmatimonadetes, Nitrospirae, Hydrogenedentes, Armatimonadetes,* and WPS2 (Fig. 2D,E), which may explain the high values of Shannon, Inverse Simpson diversity indices and evenness. More importantly, the five top abundant OTUs are affiliated mainly with *Xanthomonadales*, *Chitinophagales* and *Chloroflexi*: unknown OTU PLTA13 (6.5%), an unknown OTU (2.7%), the genera *Dokdonella* (2.3%), *Terrimons* (2.1%), and unknown *Chloroflexi* (Table S4). *Xanthomonadales* and *Chitinophagales* are known to be EPS producers^51,52^.

Members of the *Chitinophagaceae* family dominating the sludge during the third period, are aerobic heterotrophs capable of degrading organic matter and are involved in biosynthesizing and exporting extracellular polymeric substances (EPSs)^51^. Among the *Chitinophagales*, *Ferruginibacter* is among the top 10 bacterial predominant OTUs; represents 1.5% of the total reads within the third period of colonization of the SM_WWTP. Previous studies reported that *Ferruginibacter* are highly enriched in sludge and potentially associated with bio-flocculation and biofilms^53,54^ consistently supporting their suggested role in floc formation. In a recent report, Saunders et al*.,* analyzed the microbial communities in 13 Danish wastewater treatment plants in consecutive years and a single plant periodically over 6 years, using Illumina sequencing of 16S ribosomal RNA amplicons of the V4 region^4^. The plants contained a core community of 63 abundant genus-level OTUs that made up 68% of the total reads. They showed that *Chitinophagaceae* and *Comamonadaceae* represent a core bacterial members of sludge that frequently occur across many full-scale, geographically differentially located WWTPs. Hence, this study shows that structural and metabolic complexity of the ecosystem involves EPS synthetizing bacteria, which predominate over the second and the third period of the colonization process.

Our results show the existence of high proportion of longstanding prokaryotic and mostly eukaryotic persistent species in activated sludge. They may sustain the long-term ecosystem functional stability. While the abundances of bacterial OTUs decreases steadily throughout the period of sampling (Fig. S7A), the eukaryotic persistent OTUs maintain their abundances within a frame of 60–100% of the total eukaryotic reads (Fig. S7B).

In summary, we could distinguish clearly 2–3 types of population patterns within both *Bacteria* and *Eukarya* domains at various phylogenetic levels (Phylum, Order or OTU): first, r-strategists referred to as “opportunistic”, or first colonizers, are free living microorganisms, emphasize high growth rates with less diversity, typically exploiting less-crowded ecological niches and producing many offspring. Second, K-Strategists or late colonizers, are selected species described as “equilibrium”. They display traits mostly associated within flocs, close to carrying capacity of the aerobic basin and typically are strong competitors in such crowded niches. Finally, the continuous spectrum populations, which are populations withstanding the physicochemical transformations occurring in the ecosystem. They represent the persistent OTUs all over the colonization period.

## Conclusion and perspectives

Physicochemical and microbial parameters show that the wastewater microbiome is established sequentially over a long period. They start in part by heterotrophic nitrification–denitrification processes and become auto- and heterotrophic in the second phase when the flocs are well structured. The development of highly structured flocs triggered by EPS producing microorganisms, which become predominant with time, creates specific anaerobic niches triggering among other functions, the autotrophic nitrification–denitrification processes. The assemblage of various microorganisms within flocs favors multitude of metabolic interactions, which may be assessed by multi-meta-omics (meta-transcriptomics, metabolomics and meta-proteomics) technologies. Whole shotgun sequencing metagenomes of representative samples from each phase of colonization represents a first step toward understanding how the microbiota is gradually creating its living and the pattern of the main functions established over time toward the building of functionally stable ecosystem. Importantly, the onset of the nitrification process is correlated with radical changes in both prokaryotic and eukaryotic populations, leading from a planktonic mode of growth of microbial populations, to a fully functional wastewater treatment plant, associated with populations organized around floc structures.

Further characterization of the microbiota by sequencing the WWTP viriome is more than needed to have a holistic view of the microbial interactions and to explore the networks of relationships between the continuously evolving microbial community members of the SM_WWTP microbiome. Based on their presence, their richness, the abiotic factors (physicochemical parameters), and inter-taxa correlations, taxon co-occurrence or exclusion patterns, we will be able to gain more integrated understanding of microbial communities’ structure and the ecological rules guiding assembly of complex microbial communities across temporal gradients.

## Material and methods

### Wastewater treatment plant description and sampling

The SM_WWTP, is located at Blanc-Mesnil (48° 57′ 09.6″ N 2° 27′ 46.5″ E, Seine-Saint-Denis, France), was commissioned in March 2014. The SM_WWTP has treatment capacity of 50,000-m^3^ day^−1^, which can be extended to 76,500-m^3^ day^−1^ in rainy weather. It treats wastewater from a residential area of 200,000 inhabitants in the northeastern of Paris and effluents from Roissy-Charles de Gaulle International Airport. It discharges its treated effluents into the Morée stream, a 12.4 km long river, flowing into the Croult, which joins the Seine River. This river has been heavily impacted by the lack of a wastewater treatment plant since the nineteenth century. The purpose/goal of the SM-WWTP is to restore the quality of its water in accordance with the European Water Framework Directive.

This plant started de novo without any external sludge inoculation. It was filled up with potable water on March 3rd, 2014, and gradually supplied with raw wastewater. The plant performance data was made available by the wastewater facility. Water temperature, Biochemical oxygen demand (BOD), Chemical oxygen demand (COD), pH, gross flow rate discharge, total suspended solids (TSS), oxygen concentration in aerobic sludge, settling volume, dryness, total volatile suspended solids, Mohlman index, nitrogen (total Kjeldahl nitrogen (TKN), ammonia (N-NH4), nitrite (N-NO_2_ and nitrate N-NO_3_) and phosphorus concentrations were determined according to standard methods. Physicochemical parameters measurements were performed every day by the SIAAP (Syndicat Interdépartemental pour l'Assainissement de l'Agglomération Parisienne) laboratory at the entrance and the exit of the biological tank. The measurements were performed according to the French standards methods: TSS, according to NF EN 872; TKN, according to NF EN 25663; Ammonia, according to NF EN ISO 11732; nitrite and nitrate, according to NF EN ISO 13395; Orthophosphate and total P, according to NF EN ISO 6878. For the sequencing work, the sampling was done independently of physicochemical parameter measurements. A summary of important plant chemical and operational parameters is shown in Table S1.

Activated sludge samples were collected from the aerobic basin for 237 consecutive days (starting from March 18th, 2014 to October 27th). For each sampling point, 2 L of wastewater samples were directly collected at the end of the aeration tank. Collected wastewater samples were shipped refrigerated at 4 °C and analyzed within 6 h. Samples were then concentrated by centrifugation at 6,500*g* for 15 min at 4 °C. The pellets of the wastewater samples were kept at − 20 °C prior to metagenomic DNA extraction.

### DNA extraction, amplification and sequencing

DNA extraction was performed using Nucleo spin soil DNA kit (Macherey Nagel GmbH & Co. KG, Durën, Germany). DNA extracts were quantified by a spectrophotometric method using the WPA Biowave II UV/Visible spectrophotometer (Biochrom, Cambridge, UK) and a TrayCell Fibre optic micro cell (Hellma GmBH & Co. KG, Müllheim, Germany).

Prokaryotic diversity was investigated by PCR amplification of the hypervariable region V4-V5 of the SSU rDNA gene with fusion primers 515F (5′-Ion adapter–Barcode–GTGYCAGCMGCCGCGGTA-3′) and 928R (5′-Ion trP1 adapter–CCCCGYCAATTCMTTTRAGT-3′)^55^, which include a barcode and sequencing adapters. The fusion PCR method uses fusion primers to fix the Ion A adapter (5′-CCATCTCATCCCTGCGTGTCTCCGACTCAG-3′) linked to a barcode, and the Ion truncated P1 (trP1) adapter (5′-CCTCTCTATGGGCAGTCGGTGAT-3′) to the amplicons as they are generated during PCR. The PCR mix contained 1X Pfx amplification Buffer, 0.3 mM of each dNTP, 1 mM MgSO_4_, 0.3 µM of each primer, 1U Platinum Pfx DNA polymerase (Invitrogen), and 10–20 ng of template DNA in a 50 µL reaction volume. Amplification was performed as follows: 5 min at 94 °C, 30 cycles of 15 s at 94 °C, 30 s at 50 °C, 1 min at 68 °C, followed by final extension of 5 min at 68 °C. PCR products were purified using Agencourt AMPure XP magnetic beads (Beckman Coulter) according to the manufacturer’s instructions, with a bead versus amplicon ratio of 1.2, and eluted in 45 µL TE Buffer (10 mM Tris–HCl pH 8.0, 1 mM EDTA). Purified amplicons were quantified using DNA 1,000 Kit and 2,100 Bioanalyzer (Agilent Technologies), following the manufacturer’s instructions. Then, all amplicons were pre-diluted at 500 pM in molecular grade water and equimolarly pooled. The pool was then diluted at 100 pM for sequencing. Briefly, to prepare template-positive Ion Sphere Particles (ISPs) containing clonally amplified DNA by emulsion PCR, the library was diluted to 26 pM and set up on the Ion OneTouch 2 Instrument (Life Technologies) using the Ion PGM Hi-Q View OT2 Kit (Life Technologies) following the manufacturer’s instructions. These templated ISPs were purified on the Ion OneTouch ES (Life Technologies) according to the manufacturer’s instructions. Prokaryotic rDNA gene sequencing was performed on Ion Torrent Personal Genome Machine using Ion 316 Chip V2 (Life Technologies) and the Ion PGM Hi-Q View Sequencing Kit (Life Technologies) according to the manufacturer’s instructions. The Torrent suite software processed sequencing data. The software filtered out low quality and polyclonal sequence reads, and quality filtered data were exported as FastQ files.

Eukaryotic diversity was investigated by PCR amplification of the V9 hypervariable region of the SSU rRNA genes using the two primers 1389F/1510R (25 cycles in triplicate). The resulting 150 bp PCR fragments were subjected to Illumina libraries preparations that were sequenced on MiSeq platform at Genoscope (Evry, France). Although a comparison of different 18S rRNA gene targeting primers within the V4 region performed best^56^ we used the V9 barcode for the following reasons. (1) it presents a combination of advantages for addressing general questions of eukaryotic biodiversity over extensive taxonomic and ecological scales, (2) it is universally conserved in length (130 ± 4 bp) and simple in secondary structure, thus allowing relatively unbiased PCR amplification across eukaryotic lineages followed by Illumina sequencing, (3) it includes both stable and highly-variable nucleotide positions over evolutionary time frames, allowing discrimination of taxa over a significant phylogenetic depth, and (4) it is extensively represented in public reference databases across the eukaryotic tree of life, allowing taxonomic assignment amongst all known eukaryotic lineages^57^. Despite the high rarefaction coverage and the presence of sequences belonging to many major micro-eukaryotic taxa, we cannot exclude that our primer set covering the V9 region of the 18S rRNA gene may have not sufficiently mapped the diversity in particular of non-ciliate groups or minor populations in the SM_WWTPs.

### Sequence quality control and bioinformatics processing

For 18S rDNA Illumina reads, quality control began by removing adapters and primers on the whole reads and low quality nucleotides from both ends, and then we continued the next steps using the longest sequence without adapters or low quality bases. Reads shorter than 30 nucleotides after trimming and read pairs that come from the low-concentration spike-in library of Illumina PhiX Control were discarded. This policy allows submission of high quality data (without contamination) in order to interrogate databases and to improve subsequent analysis. Overlapping 18S rDNA paired end reads were merged with pear v0.9.11 (https://github.com/easybuilders/easybuild-easyconfigs/pull/6653/files). For 16S rDNA reads, we used cutadapt 1.12 (https://gensoft.pasteur.fr/docs/cutadapt/1.12/guide.html) to remove adapters, primers, and discarded reads with low quality nucleotides (when quality value is < 20).

Dereplicated 18S and 16S rDNA reads were independently clustered with swarm 2.1.12 (https://bioweb.pasteur.fr/packages/pack@swarm@2.1.12), using a distance cutoff of 3%, and singleton OTUs were removed. Chimeric sequences were detected with VSEARCH (https://github.com/torognes/vsearch), and removed for subsequent analyses.

16S and 18S rDNA sequence analyses were continued using the FROGS pipeline “Find, Rapidly, OTUs with Galaxy Solution” (https://frogs.toulouse.inrae.fr/). Taxonomic affiliation of 16S and 18S rDNA reads was performed with BLAST 2.6 on SILVA_132_16S and SILVA_132_18S databases respectively. A Biological Observation Matrix file (BIOM) comprising both abundance and taxonomy, was generated and imported into R (version 3.5.2) for statistical analysis.

### Statistical analyses

Alpha diversity estimators were computed using the Phyloseq software package^58^, which is an integrated algorithm into FROGS pipeline. Briefly, calculation of community richness and alpha-diversity indices (e.g. Shannon–Weiner and InvSimpson) was done using BIOM files. In this paper, we show only the evolution of Shannon–Weiner and inverse Simpson indices over time. A hierarchically clustered heat-map was generated using the top 100 microbial OTUs respectively for prokaryotes and eukaryotes respectively from the 23 time-series SM_WWTP samples. We used Bray–Curtis dissimilarity to quantify the differences between OTUs and species among the time series SM_WWTP samples.

### Principal component and canonical component analyses

In order to get insights into the relationships between WWTP time series samples, centered, scaled, principal component analyses (PCoA) were implemented on 16S rDNA and 18S rDNA sequence tags datasets respectively using ADE4 R package^59^. Considering the dispersion in the total number of OTUs reads identified in each sample, OTUs abundances were scaled by dividing the number of reads of each OTU in a given sample by the sum of total reads for the same sample. We considered only OTUs that exceeded 1% in at least one sample for the analysis. We kept 117 OTUs of 16S rDNA and 98 OTUs of 18S rDNA for subsequent analyses. We took into account OTUs variable distributions represented by axes accounting for the highest percent of the total variance. To get insight into the relationships between physicochemical and bacterial communities evolution, we implemented canonical correspondence Analysis (CCA) using vegan R package. PCoA and CCA. For both PCoA and CCA, figures were visualized using ggplot2.

### Originality-significance-statement

The originality of the study resides in its 8-month sequential follow-up of both bacterial and micro-eukaryotic community colonization of a newly constructed wastewater treatment plant (WWTP) seeded by raw water microorganisms rather than sludge from a different WWTP as is commonly done. To the best of our knowledge, we do not know of any study that analyzes microbial colonization kinetics from the beginning of operations up to the establishment of a complex environmental microbial ecosystem. It leads to a holistic view of the wastewater microbiome structure, population dynamics and the kinetics of its establishment, thanks to high throughput sequencing technology and sampling of a newly constructed WWTP over time. We were able to distinguish mainly three populations patterns. The first colonizers, represented by unstructured populations with high growth rates, typically exploit less-crowded ecological niches and produce many offspring; the second colonizers, that take over from the first colonizers form a structured population, we call K-Strategists or equilibrium strategists. They display traits associated with living at densities close to the carrying capacity of the milieu; and finally, continuous spectrum populations, that may represent the backbone of microbial communities. This group shows strong traits of longevity and may guarantee microbial community functioning, due to the buffering capacity they provide to the ecosystem to help resist environmental change.

## Supplementary information

Supplementary Information 1.

Supplementary Information 2.

## Data Availability

Raw data for relative abundance of both eukaryotic and bacterial communities at the different taxonomic levels will be made available and provided on reasonable request. Sequences reported in this study were deposited in EMBL databases (https://www.ebi.ac.uk/) under accession numbers ERX4094207-ERX4094229; and ERR4106944-ERR4106967 for eukaryotic sequences and ERS4556742 to ERS4556765 for bacterial sequences.

## References

[1] Grady CPL, Daigger GT, Love NG (2011). Biological Wastewater Treatment.

[2] Wagner M, Loy A (2002). Bacterial community composition and function in sewage treatment systems. Curr. Opin. Biotechnol..

[3] Ju F, Zhang T (2015). Bacterial assembly and temporal dynamics in activated sludge of a full-scale municipal wastewater treatment plant. Isme J..

[4] Saunders AM, Albertsen M, Vollertsen J, Nielsen PH (2016). The activated sludge ecosystem contains a core community of abundant organisms. Isme J..

[5] Matsunaga K, Kubota K, Harada H (2014). Molecular diversity of eukaryotes in municipal wastewater treatment processes as revealed by 18S rRNA gene analysis. Microbes Environ..

[6] Chouari R (2017). Eukaryotic molecular diversity at different steps of the wastewater treatment plant process reveals more phylogenetic novel lineages. World J. Microbiol. Biotechnol..

[7] Matsubayashi M, Shimada Y, Li YY, Harada H, Kubota K (2017). Phylogenetic diversity and in situ detection of eukaryotes in anaerobic sludge digesters. PLoS ONE.

[8] Cohen Y (2019). Bacteria and microeukaryotes are differentially segregated in sympatric wastewater microhabitats. Environ. Microbiol..

[9] Flowers JJ, Cadkin TA, McMahon KD (2013). Seasonal bacterial community dynamics in a full-scale enhanced biological phosphorus removal plant. Water Res..

[10] Zhang B (2018). Seasonal bacterial community succession in four typical wastewater treatment plants: correlations between core microbes and process performance. Sci. Rep..

[11] Stein LY, Arp DJ, Hyman MR (1997). Regulation of the synthesis and activity of ammonia monooxygenase in *Nitrosomonas europaea* by altering pH to affect NH(inf3) availability. Appl. Environ. Microbiol..

[12] Chain P (2003). Complete genome sequence of the ammonia-oxidizing bacterium and obligate chemolithoautotroph *Nitrosomonas europaea*. J. Bacteriol..

[13] Pedersen H, Dunkin KA, Firestone MK (1999). The relative importance of autotrophic and heterotrophic nitrification in a conifer forest soil as measured by 15N tracer and pool dilution techniques. Biogeochemistry.

[14] White JP, Johnson GT (1982). Aflatoxin production correlated with nitrification in *Aspergillus flavus* group species. Mycologia.

[15] Lang E (1986). Fungi of a forest soil nitrifying at low pH values. FEMS Microbiol. Ecol..

[16] Stroo HF (1986). Heterotrophic nitrification in an acid forest soil and by an acid-tolerant fungus. Appl. Environ. Microbiol..

[17] Guest R, Smith D (2007). Isolation and screening of fungi to determine potential for ammonia nitrogen treatment in wastewater. J. Environ. Eng. Sci..

[18] Hultberg M, Bodin H (2017). Fungi-based treatment of brewery wastewater—biomass production and nutrient reduction. Appl. Microbiol. Biotechnol..

[19] Liu Z (2016). *Paecilomyces variotii*: a fungus capable of removing ammonia nitrogen and inhibiting ammonia emission from manure. PLoS ONE.

[20] Moir JW, Crossman LC, Spiro S, Richardson DJ (1996). The purification of ammonia monooxygenase from *Paracoccus denitrificans*. FEBS Lett..

[21] Jetten MS (1997). Novel principles in the microbial conversion of nitrogen compounds. Antonie Van Leeuwenhoek.

[22] Beller HR (2006). The genome sequence of the obligately chemolithoautotrophic, facultatively anaerobic bacterium *Thiobacillus denitrificans*. J. Bacteriol..

[23] Joo HS, Hirai M, Shoda M (2007). Improvement in ammonium removal efficiency in wastewater treatment by mixed culture of *Alcaligenes faecalis* no. 4 and L1. J. Biosci. Bioeng..

[24] Su JJ, Liu BY, Liu CY (2001). Comparison of aerobic denitrification under high oxygen atmosphere by *Thiosphaera pantotropha* ATCC 35512 and *Pseudomonas stutzeri* SU2 newly isolated from the activated sludge of a piggery wastewater treatment system. J. Appl. Microbiol..

[25] Patureau D (2001). Combined phosphate and nitrogen removal in a sequencing batch reactor using the aerobic denitrifier, *Microvirgula aerodenitrificans*. Water Res..

[26] Kim M (2008). Aerobic denitrification of *Pseudomonas putida* AD-21 at different C/N ratios. J. Biosci. Bioeng..

[27] Zhao B, He YL, Hughes J, Zhang XF (2010). Heterotrophic nitrogen removal by a newly isolated *Acinetobacter calcoaceticus* HNR. Bioresour. Technol..

[28] Willems A, Rosenberg E (2014). The family *Comamonadaceae*. The prokaryotes: Alphaproteobacteria and betaproteobacteria.

[29] Wu Y, Shukal S, Mukherjee M, Cao B (2015). Involvement in denitrification is beneficial to the biofilm lifestyle of *Comamonas testosteroni*: a mechanistic study and its environmental implications. Environ. Sci. Technol..

[30] Rosch C, Mergel A, Bothe H (2002). Biodiversity of denitrifying and dinitrogen-fixing bacteria in an acid forest soil. Appl. Environ. Microbiol..

[31] Bartossek R, Nicol GW, Lanzen A, Klenk HP, Schleper C (2010). Homologues of nitrite reductases in ammonia-oxidizing archaea: diversity and genomic context. Environ. Microbiol..

[32] Cabello P, Roldan MD, Moreno-Vivian C (2004). Nitrate reduction and the nitrogen cycle in *archaea*. Microbiology.

[33] Shoun H, Kim DH, Uchiyama H, Sugiyama J (1992). Denitrification by fungi. FEMS Microbiol. Lett..

[34] Hayatsu M, Tago K, Saito M (2008). Various players in the nitrogen cycle: Diversity and functions of the microorganisms involved in nitrification and denitrification. Soil Sci. Plant Nutr..

[35] Pelmont, J. *Biodégradations et métabolismes: les bactéries pour les technologies de l'environnement* (ed. Les Ulis EDP Sciences DL) (Coll. Grenoble Sciences, 2005).

[36] Kobayashi M (1996). Denitrification, a novel type of respiratory metabolism in fungal mitochondrion. J. Biol. Chem..

[37] Glud RN (2009). Nitrogen cycling in a deep ocean margin sediment (Sagami Bay, Japan). Limnol. Oceanogr..

[38] Dolinsek J, Lagkouvardos I, Wanek W, Wagner M, Daims H (2013). Interactions of nitrifying bacteria and heterotrophs: identification of a Micavibrio-like putative predator of *Nitrospira* spp. Appl Environ Microbiol.

[39] Shaw LJ (2006). *Nitrosospira* spp. can produce nitrous oxide via a nitrifier denitrification pathway. Environ. Microbiol..

[40] Medhi K, Singhal A, Chauhan DK, Thakur IS (2017). Investigating the nitrification and denitrification kinetics under aerobic and anaerobic conditions by *Paracoccus denitrificans* ISTOD1. Biores. Technol..

[41] Khanichaidecha W, Ratananikom ANK, Eamrat R, Kazama F (2019). Heterotrophic nitrification and aerobic denitrification using pure-culture bacteria for wastewater treatment. J. Water Reuse Desalin..

[42] Amann RI, Ludwig W, Schleifer KH (1995). Phylogenetic identification and in situ detection of individual microbial cells without cultivation. Microbiol. Rev..

[43] Khanichaidecha W, Nakaruk A, Ratananikom K, Eamrat R, Kazama F (2019). Heterotrophic nitrification and aerobic denitrification using pure-culture bacteria for wastewater treatment. J. Water Reuse Desalin..

[44] Zeng W, Li B, Wang X, Bai X, Peng Y (2016). Influence of nitrite accumulation on "Candidatus Accumulibacter" population structure and enhanced biological phosphorus removal from municipal wastewater. Chemosphere.

[45] Ganesh S (2015). Size-fraction partitioning of community gene transcription and nitrogen metabolism in a marine oxygen minimum zone. Isme J..

[46] Flemming HC, Wingender J (2001). Relevance of microbial extracellular polymeric substances (EPSs)—part i: structural and ecological aspects. Water Sci. Technol..

[47] Wingender J, Thomas RN, Flemming H-C (1999). Microbial Extracellular Polymeric Substances: Characterization, Structure and Function.

[48] More TT, Yadav JS, Yan S, Tyagi RD, Surampalli RY (2014). Extracellular polymeric substances of bacteria and their potential environmental applications. J. Environ. Manag..

[49] Naessens M, Cerdobbel A, Soetaert W, Vandamme EJ (2005). Leuconostoc dextransucrase and dextran: production, properties and applications. J. Chem. Technol. Biotechnol..

[50] Rehm B (2009). Microbial Production of Biopolymers and Polymer Precursors: Applications and Perspectives.

[51] Liu T, Mao YJ, Shi YP, Quan X (2017). Start-up and bacterial community compositions of partial nitrification in moving bed biofilm reactor. Appl. Microbiol. Biotechnol..

[52] Sutherland IW (2001). Microbial polysaccharides from Gram-negative bacteria. Int. Dairy J..

[53] Wang X, Hu M, Xia Y, Wen X, Ding K (2012). Pyrosequencing analysis of bacterial diversity in 14 wastewater treatment systems in China. Appl. Environ. Microbiol..

[54] Faust L (2015). Characterization of the bacterial community involved in the bioflocculation process of wastewater organic matter in high-loaded MBRs. Appl. Microbiol. Biotechnol..

[55] Wang Y, Qian PY (2009). Conservative fragments in bacterial 16S rRNA genes and primer design for 16S ribosomal DNA amplicons in metagenomic studies. PLoS ONE.

[56] Hugerth LW (2014). Systematic design of 18S rRNA gene primers for determining eukaryotic diversity in microbial consortia. PLoS ONE.

[57] De Vargas C (2015). Eukaryotic plankton diversity in the sunlit ocean. Science.

[58] McMurdie PJ, Holmes S, Watson M (2013). phyloseq: an R package for reproducible interactive analysis and graphics of microbiome census data. PLoS ONE.

[59] Thioulouse J (1989). Statistical analysis and graphical display of multivariate data on the Macintosh. Comput. Appl. Biosci..

