# Supplementary material for: Colonization kinetics and implantation follow-up of the sewage microbiome in an urban wastewater treatment plant
Source: Sci Rep. 2020 Jul 15;10:11634. doi: 10.1038/s41598-020-68496-z (PMC7363871; doi:10.1038/s41598-020-68496-z)
Supplement: Supplementary file 1 — Supplementary Information 1. [file 41598_2020_68496_MOESM1_ESM.pdf]

Colonization kinetics and implantation follow-up of the sewage microbiome in an urban wastewater treatment plant

Loïc Morin[1], Anne Goubet[2], Céline Madigou[2], Jean-Jacques Pernelle[2], Karima Palmier[1], Karine Labadie[3], Arnaud Lemainque[3], Ophélie Michot[4], Lucie Astoul[4], Paul Barbier[5], Jean-Luc Almayrac[4], and Abdelghani Sghir[5*]

[1]Institut de Biologie Intégrative de la Cellule, Université Paris Saclay, 91405 Orsay Cedex, France.

[2]Université Paris-Saclay, INRAE, PROSE, 92761, Antony, France.

[3]Genoscope, Institut de biologie François-Jacob, CEA, Université Paris-Saclay, F-91057 Evry, France.

[4]Laboratoire SIAAP Site Seine Amont, Usine Marne Aval, 100 rue de la Plaine 93160 Noisy-Le-Grand, France.

[5*]Génomique métabolique, Genoscope, Institut de Biologie François Jacob, CEA, CNRS, Université d'Evry, Université Paris-Saclay, 91057, Evry, France.

***Correspondence and requests for materials should be addressed to Abdelghani Sghir**

Email: sghir@genoscope.cns.fr

Competing interests: The authors declare no competing interests.

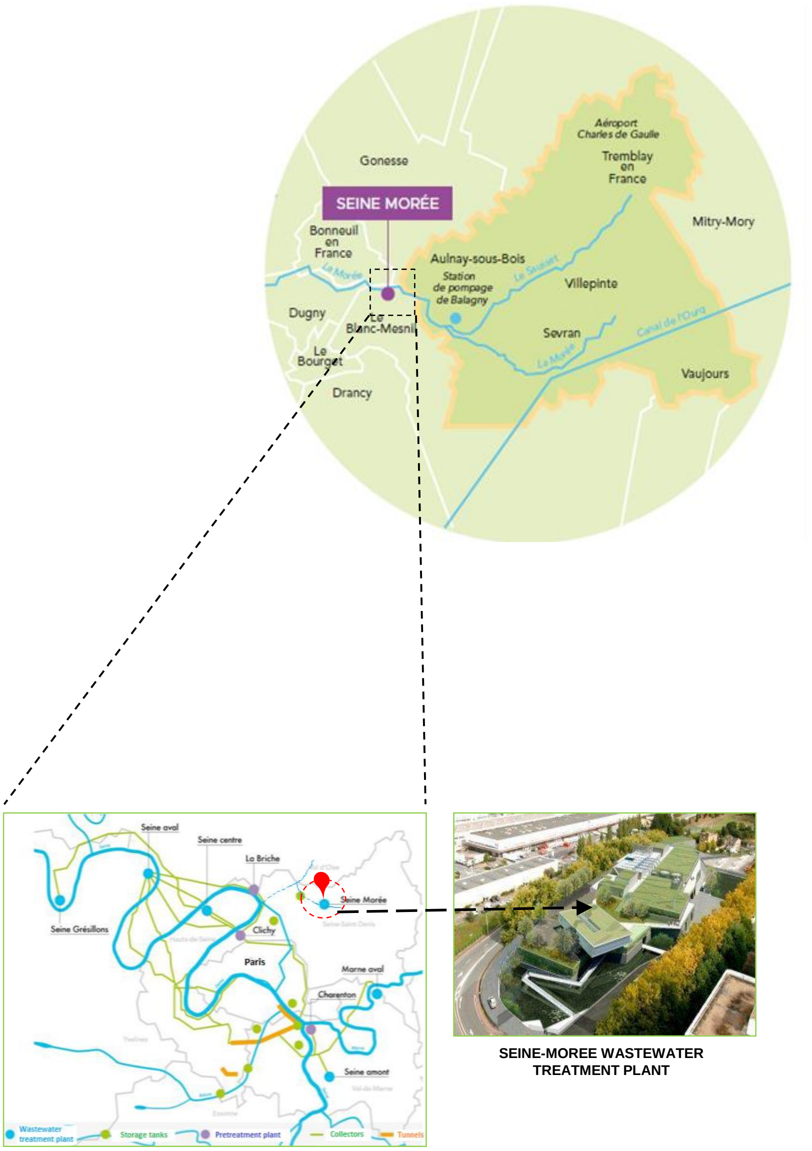

Fig. S1. SM_WWTP geolocalization

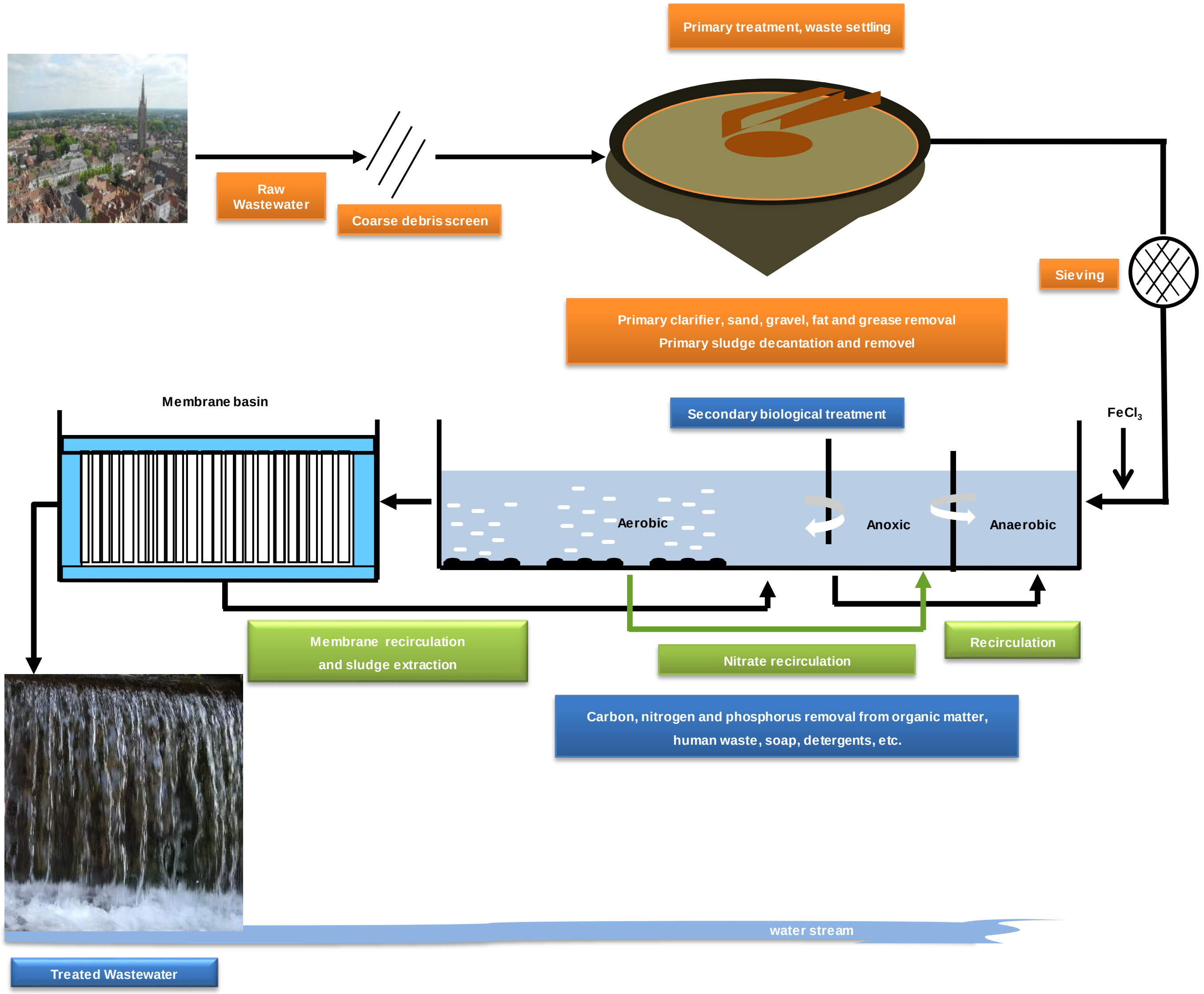

Fig. S2. SM_WWTP plant description

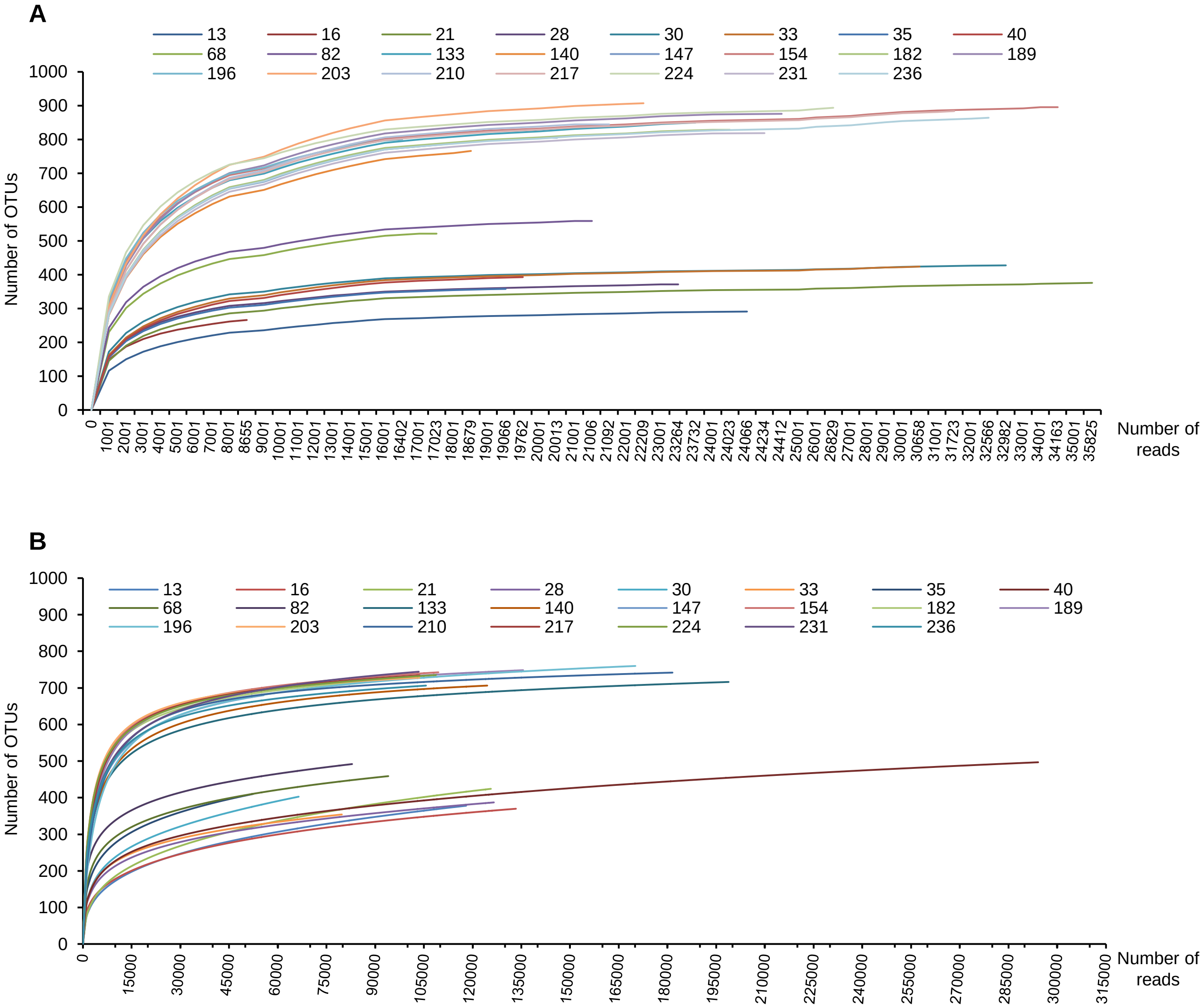

Fig. S3. Bacterial and eukaryotic rarefaction curves describing microbial richness of the time series SM_WWTP samples. Rarefaction curves describing bacterial **(A)** and eukaryotic **(B)** richness and of the 23 times series SM_WWTP samples.

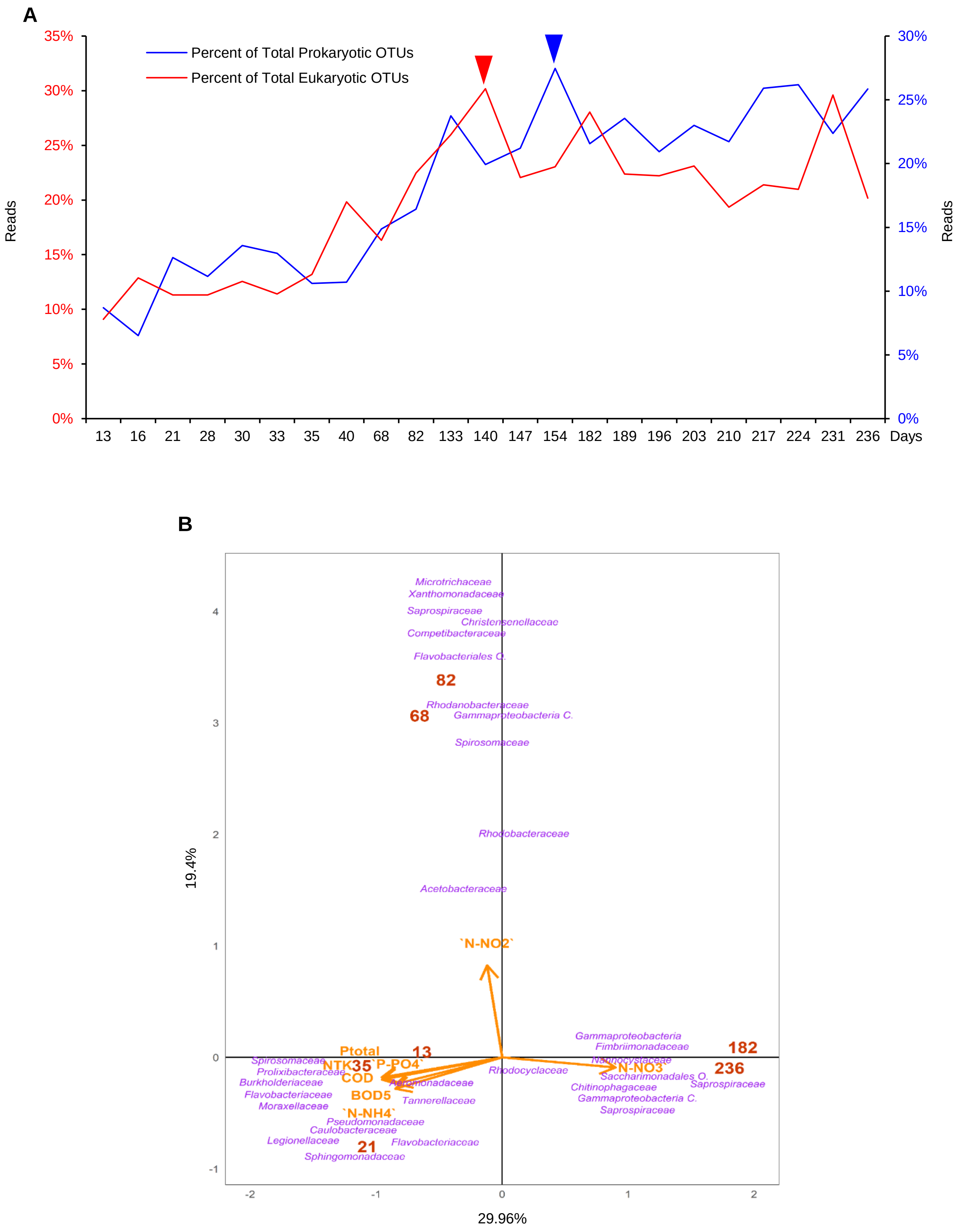

Fig. S4. (**A**) OTUs colonization kinetics within the aerobic basin of SM_WWTP. Scale colors: blue represent bacterial OTUs and red, Eukaryotic OTUs. (**B**) Canonical correspondence analysis (CCA) describing the relationship between bacterial community compositions and physicochemical properties of the time series wastewater samples.

A

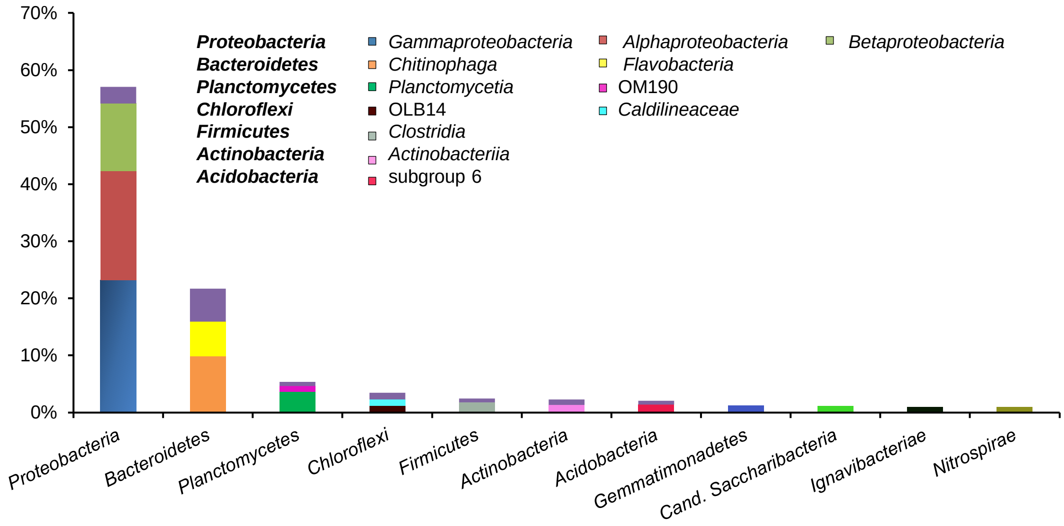

B

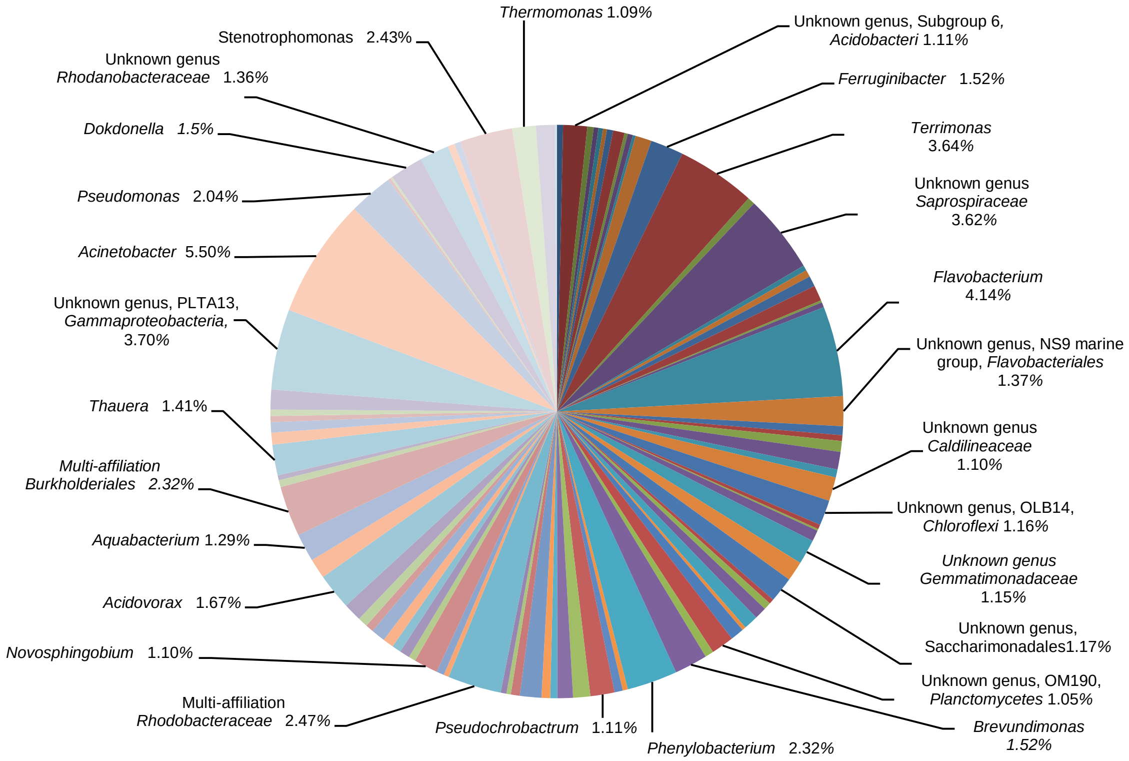

Fig. S5. Global relative abundance average (% of reads) of the bacterial phyla and genera within the 23 time series SM_WWTP samples. (**A**) Relative abundance average (%) of **bacterial phyla** reads (phyla with ≥ 0.9% of total reads). Each bar includes subphyla displayed with different colors. The top violet color represent the sum of the remaining subphyla in the corresponding phylum. For each of the first seven bars from bottom to top, the bars are representing subphyla, (**B**) Relative abundance average (%) of **bacterial genera reads**. Genera were filtered for those that display an abundance rate ≥ 1% in at least one of the 23 samples. Unaffiliated sequences to known genera, depicted as "Unknown genus" were grouped together within the nearest described taxonomic rank, i.e. family, order, or class. The same applies to those depicted as "Multi-affiliation" sequences.

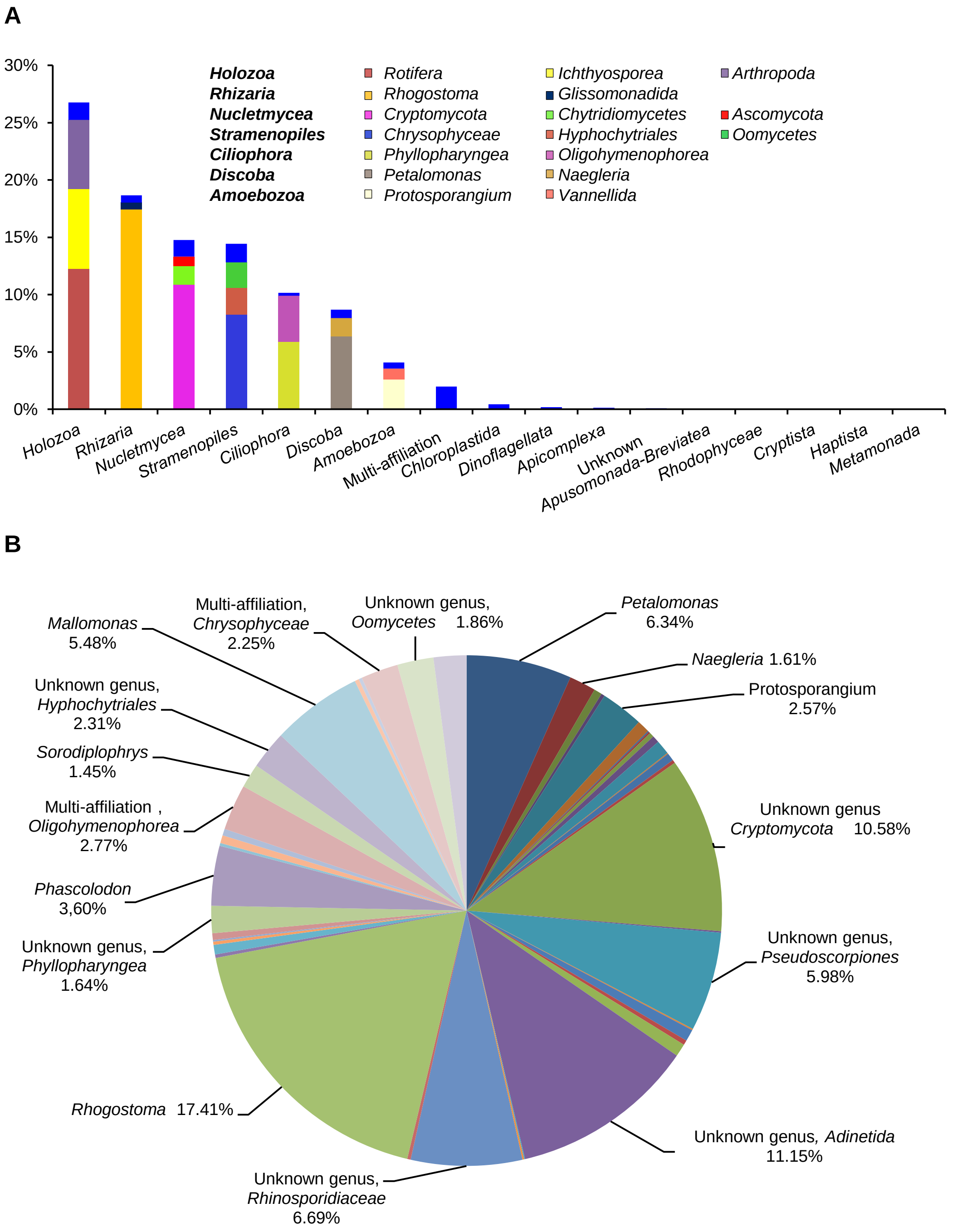

Fig. S6. Global relative abundance average (% of reads) of the eukaryotic phyla and genera within the 23 time-series SM_WWTP samples. (A) Relative abundance average (%) of **eukaryotic phyla** reads. Each bar includes subphyla and/or genera displayed with different colors. The top blue color represent the sum of the remaining subphyla in the corresponding phylum. From bottom to top, the bars represent subphyla. (**B**) Relative abundance average (%) of **eukaryotic genera** reads. Genera were filtered for those that display an abundance rate ≥ 1% in at least one of the 23 samples. Unaffiliated sequences depicted as "Unknown genus" were grouped together within the nearest known taxonomic rank, i.e. family, order, or class. The same applies to those depicted as "Multi-affiliation" sequences.

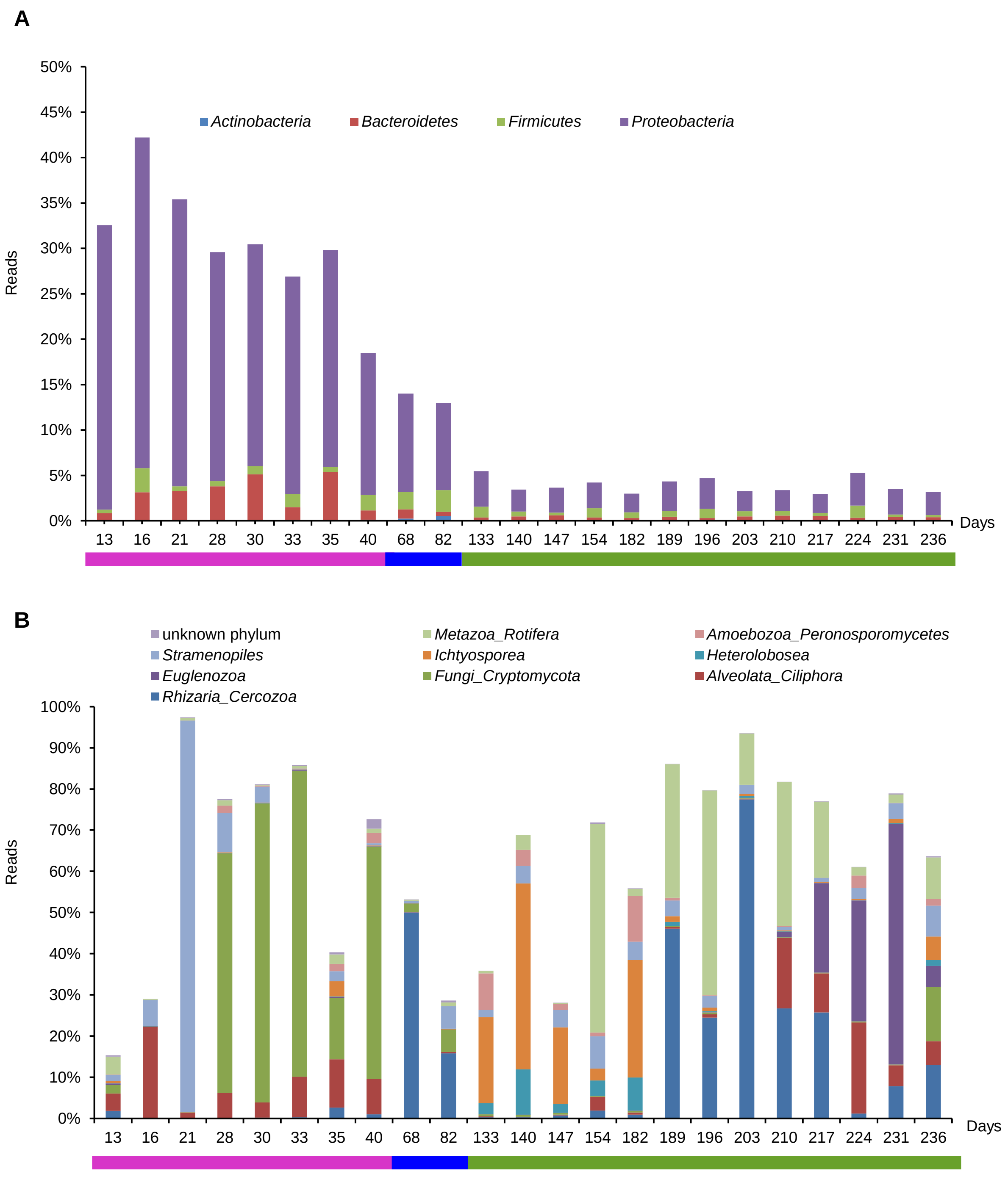

Fig. S7. Relative abundance (%) and kinetics of persistent bacterial (**A**) and eukaryotic (**B**) phyla of the 23 time-series SM_WWTP samples. Colored lines delimit the three periods of physicochemical parameters and microbiota evolution: Pink represent the first period, blue, the intermediate period, and green represent the third period.
