# Supplementary material for: Colonization kinetics and implantation follow-up of the sewage microbiome in an urban wastewater treatment plant
Source: Sci Rep. 2020 Jul 15;10:11634. doi: 10.1038/s41598-020-68496-z (PMC7363871; doi:10.1038/s41598-020-68496-z)
Supplement: Supplementary file 2 — Supplementary Information 2. [file 41598_2020_68496_MOESM2_ESM.pdf]

Colonization kinetics and implantation follow-up of the sewage microbiome in an urban wastewater treatment plant

Loïc Morin[1], Anne Goubet[2], Céline Madigou[2], Jean-Jacques Pernelle[2], Karima Palmier[1], Karine Labadie[3], Arnaud Lemainque[3], Ophélie Michot[4], Lucie Astoul[4], Jean-Luc Almayrac[4], and Abdelghani Sghir[3*]

[1]Institut de Biologie Intégrative de la Cellule, Université Paris Saclay, 91405 Orsay Cedex, France.

[2]Université Paris-Saclay, INRAE, PROSE, 92761, Antony, France.

[3*]Génomique métabolique, Genoscope, Institut de Biologie François Jacob, CEA, CNRS, Université d'Evry, Université Paris-Saclay, 91057, Evry, France.

[4]Laboratoire SIAAP Site Seine Amont, Usine Marne Aval, 100 rue de la Plaine 93160 Noisy-Le-Grand, France.

***Correspondence and requests for materials should be addressed to AS.** Email: sghir@genoscope.cns.fr

Table S1. The SM_WWTP physicochemical performances after stabilization. This Table shows the results of the plant's performance calculated from the daily measurements at the entry and exit of the WWTP over a period of one year immediately after the start up of the plant.

Parameters	Effluent concentration (mg L^{-1})	Efficiency (%)
TSS	1.10 ± 0.58	99.7 ± 0.2
COD	13.3 ± 8.6	98.1 ± 1.3
BOD	1.3 ± 1.1	99.6 ± 0.4
TKN	1 ± 0.9	98.8 ± 1.1
N-NH_4	0.35 ± 0.81	99.3 ± 1.3
NGL	8.49 ± 2.77	88.7 ± 3.8
Total P	0.23 ± 0.32	97.5 ± 3.6

TSS: Total suspended solids; COD: Chemical oxygen demand; BOD; Biochemical oxygen demand; TKN: Total Kjeldahl Nitrogen; N-NH_4: Ammonia nitrogen; NGL: Global Nitrogen (or Total Nitrogen); Total P: Total phosphorus.

Table S2. The ten predominant OTUs over the first period (13-40 days) of the SM_WWTP colonization.

OTU number	Order	Genera	Average (% of reads)
OTU_1	*Pseudomonadales*	*Acinetobacter*	13.4 ± 0.06
OTU_3	*Caulobacterales*	*Phenylobacterium*	5.7 ± 0.03
OTU_5	*Burkholderiales*	*Acidovorax*	4.2 ± 0.02
OTU_4	*Xanthomonadales*	*Stenotrophomonas*	4.0 ± 0.02
OTU_9	*Burkholderiales*	*Aquabacterium*	3.2 ± 0.03
OTU_10	*Flavobacteriales*	*Flavobacterium*	3.0 ± 0.03
OTU_27	*Caulobacterales*	*Brevundimonas*	2.5 ± 0.01
OTU_42	*Pseudomonadales*	*Acinetobacter*	2.3 ± 0.02
OTU_20	*Pseudomonadales*	*Pseudomonas*	2.3 ± 0.02
OTU_18	*Xanthomonadales*	*Stenotrophomonas*	1.8 ± 0.01

Table S3. The ten predominant OTUs over the second period (40-133 days) of the SM_WWTP colonization.

OTU number	Order	Genera	Average (% of reads)
OTU_11	*Xanthomonadales*	*Thermomonas*	13.8 ± 2.10
OTU_83	*Competibacterales*	Candidatus Competibacter	2.9 ± 0.08
OTU_74	*Xanthomonadales*	*Arenimonas*	2.0 ± 0.47
OTU_1	*Pseudomonadales*	*Acinetobacter*	2.2 ± 0.41
OTU_108	*Flavobacteriales*	Uknown genus	1.9 ± 0.84
OTU_152	*Microtrichiales*	Uknown genus	1.6 ± 0.46
OTU_43	*Burkholderiales*	Multi-affiliation	1.4 ± 0.06
OTU_142	Flavobacteriales	Unknown genus	1.3 ± 0.47
OTU_84	*Xanthomonadales*	*Pseudoxanthomonas*	1.3 ± 0.13
OTU_191	*Clostridiales*	*Christensenellaceae* R-7 group	1.1 ± 0.28

Table S4. The ten predominant OTUs over the third period (133-236 days) of SM_WWTP colonization.

OTU number	Order	Genera	Average<br>(% of reads)
OTU_2	*Xanthomonadales*	PLTA13	6.5 ± 2.55
OTU_6	*Chitinophagales*	Unknown genus	2.7 ± 1.03
OTU_7	*Xanthomonadales*	*Dokdonella*	2.3 ± 1.10
OTU_22	*Chitinophagales*	*Terrimonas*	2.1 ± 2.04
OTU_15	*Chloroflexi*	unknown genus	1.8 ± 1.13
OTU_13	*Chitinophagales*	*Terrimonas*	1.7 ± 1.95
OTU_12	*Chitinophagales*	*Ferrugibacter*	1.5 ± 1.71
OTU_14	*Acidobacteriales*	Multi-affiliation	1.5 ± 0.79
OTU_19	*Rhodobacterales*	Multiaffiliatioon	1.3 ± 0.85
OTU_8	*Rhodocyclales*	*Thauera*	1.2 ± 0.56
